# Transglutaminase 3 Reduces the Severity of Psoriasis in Imiquimod-Treated Mouse Skin

**DOI:** 10.3390/ijms21051566

**Published:** 2020-02-25

**Authors:** Maria Cristina Piro, Alessandra Ventura, Artem Smirnov, Andrea Saggini, Anna Maria Lena, Alessandro Mauriello, Luca Bianchi, Gerry Melino, Eleonora Candi

**Affiliations:** 1 Department of Experimental Medicine, TOR, University of Rome “Tor Vergata”, 00133 Rome, Italy; piro@med.uniroma2.it (M.C.P.); art.smirnow@gmail.com (A.S.); andreasaggini@gmail.com (A.S.); lena@med.uniroma2.it (A.M.L.); alessandro.mauriello@uniroma2.it (A.M.);; 2 Dermatology Unit, Department of Biotechnological and Applied Clinical Science, University of L’Aquila, IT-67100 L’Aquila, Italy; alessandraventura@live.it; 3 Dermatology Unit, Department of Systems Medicine, University of Rome Tor Vergata, 00133 Rome, Italy; luca.bianchi@uniroma2.it; 4 Medical Research Council, University of Cambridge, Cambridge CB21QP, UK; 5 IDI-IRCCS, Biochemistry laboratory, 00167 Rome, Italy

**Keywords:** transglutaminase 2, transglutaminase 3, psoriasis, imiquimod, inflammation, skin, TG3KO, TG2KO

## Abstract

Four transglutaminase (TG) isoforms have been detected in epidermal keratinocytes: TG1, TG2, TG3, and TG5. Except for TG1 and TG3, their contribution to keratinocyte development and structure remains undefined. In this paper, we focused on the roles of TG2 and TG3 in imiquimod-induced psoriasis in mouse skin. We evaluated the severity of psoriasis markers in the skin of imiquimod-treated TG3 null and TG2 null mice. Our results showed that compromised TG3KO mouse skin was more responsive than WT or TG2KO mouse skin to the action of the pro-inflammatory drug imiquimod.

## 1. Introduction

Transglutaminases (TGs) are a class of Ca^+^-dependent enzymes that catalyse protein crosslinking by the formation of a covalent bond between an amine donor, often a peptide-bound lysine, and the γ-carboxamide of a glutamine side chain of selected proteins. Originally described in 1959 in guinea pig liver, TGs play a central role in the creation and reinforcement of epithelial barriers, while at the extracellular level, they consolidate the extracellular matrix or stabilize fibrin clots [1,2]. The TG family now encompasses nine members, and TG1, TG3, and TG5 are expressed in the epidermis and are known to be active in the formation of the cornified cell envelope (CE), a highly insoluble lipid-protein structure that is present in terminally differentiated keratinocytes and is necessary for the barrier function of the epidermis [3]. In suprabasal keratinocytes proteins such as loricrin, filaggrin, involucrin, and trichohyalin, small proline-rich proteins and keratins are crosslinked by TG1, TG3, and TG5 [4,5]. 

Because of their critical and primary role in the formation of cutaneous barriers, defects in the genes for TG1, TG3, and TG5 cause important impacts on the integrity and function of the epidermis and might also manifest as congenital skin or hair diseases. *Tgm1* mutations in humans are the molecular basis of autosomal recessive lamellar ichthyosis [6,7], a skin disorder that is characterized by hyperkeratosis and impaired cornification of the epidermis. TG1KO mouse are neonatal lethal and show an ichthyosis–like phenotype [8]. Acral peeling skin syndrome is derived from TGM5 impairing mutations [9]. In this disorder, the stratum corneum and granulosum of the epidermis are critically disjointed [10]. TG5KO mouse have not been developed so far. Finally, a mutation in the *TGM3* gene seems to be responsible for human uncombable hair syndrome (UHS) [11], whereas ablation of the TG3 gene in mice causes abnormal hair fiber morphogenesis [12]. 

TG3 is expressed in the hair follicle and in the granular and spinous layers of the epidermis [13,14]. In addition to its involvement in hair development, TG3 also contributes significantly to the proper formation of the cutaneous barrier because TG3KO mice skin displays increased permeability and susceptibility to the hapten fluorescein isothiocyanate (FITC) [15]. Moreover, our recent murine study demonstrated the importance of TG3 crosslinking activity in protecting skin from UV damage [16]. Impaired epidermal structure in TG3KO animals failed to shield against UVB radiation which thus penetrates deeper through the lower layers of epidermal keratinocytes, reaching the dermis and provoking high levels of DNA damage and apoptosis. 

A fourth enzyme, TG2, is widely diffused in various tissues and cell types. In the skin, TG2 is localized in dermal fibroblasts, and it is detected in basal keratinocytes only in specialized conditions such as wound healing and repair [17,18]. However, according to other authors, TG2 is constitutively expressed in all epidermal layers [19]. TG2 is thought to play a minor role in keratinocyte CE assembly, since mice lacking TG2 are viable, phenotypically normal, and do not show skin barrier defects [20,21,22]. TG2 also shows other mutually exclusive, peculiar enzymatic properties such as GTPase [23], protein disulfide isomerase (PDI) [24], and protein kinase activities [25]. In the extracellular space, TG2 acts as protein scaffold to assemble ECM (extracellular matrix) proteins or as a cellular signalling intermediate when it is attached to the cell membrane [26]. TG2 performs a wide range of cellular and tissue functions, often with opposite outcomes, such as proliferation, apoptosis, differentiation, or autophagy [27]. Although TG2 does not seem to play a central role in the epidermis, it is well known to have powerful pro-inflammatory actions, not only in autoimmune pathologies such as celiac or Crohn’s disease, but also in chronic inflammatory diseases involving fibrosis generation in the lung, liver, or kidneys [28,29,30,31,32]. Moreover, TG2 activates the transcription factor NF-kappaB, which positively controls various genes involved in immune and inflammatory responses and in the production of pro-inflammatory cytokines and chemokines [33,34]. In this regard, TG2 has been recently proposed as a mediator of the epidermal inflammatory response to UV radiation. In WT keratinocytes, UV irradiation activates TG2, which in turn induces the expression of inflammatory cytokines such as TNF-α and IL6. Upon UV exposure, TG2 null mice display decreased skin inflammation compared to that of WT mice [19]. 

Psoriasis is an autoimmune and inflammatory skin disease for which the complex genetic architecture has not yet been well defined [35]. Psoriasis affects 2%-3% of the global population [36] and can produce various phenotypes with different levels of severity and skin barrier impairment. The most common phenotype is psoriasis vulgaris, in which abnormal keratinocyte differentiation leads to the formation of skin plaques with a thickened epidermis (acanthosis), retention of nuclei in the stratum corneum (parakeratosis), and inflammatory infiltration in the dermis and epidermis [35,37] 

Although TG1, TG2, TG3, and TG5 play pivotal roles both in skin barrier structure and/or inflammatory processes, the contribution of their activities as causes and effects of psoriatic disease remains elusive. Topical application of imiquimod, a ligand of TLR7 and TLR8, induces a psoriasis-like skin inflammation with typical microscopic and macroscopic psoriatic markers in mice, and is the most commonly used psoriasis animal model [38].

We aimed to evaluate whether ablation of the TGM2 or TGM3 gene affected the severity of psoriasis markers in the context of imiquimod-treated TG3 and TG2 null mice. Our data showed that, at the microscopic but not macroscopic level, compromised TG3KO mouse skin was more responsive than WT or TG2KO mice skin to the action of the pro-inflammatory drug imiquimod. In contrast, TG2 was not relevant to imiquimod-induced psoriatic inflammation.

## 2. Results

### 2.1. Both TGM1 and TGM3 Are Upregulated in Skin from Patients with Psoriasis

**Figure 1:**
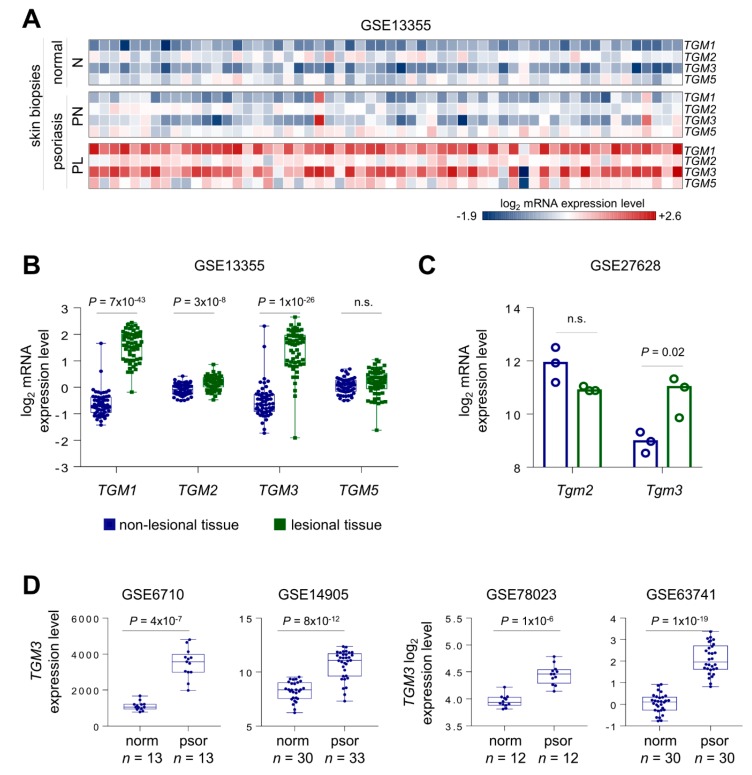
*TGM1* and *TGM3* are upregulated in skin samples from psoriasis patients. **(A)** Heat map showing the expression levels of *TGM1*, *TGM2*, *TGM3*, and *TGM5* in normal human skin (N), uninvolved non-lesional (PN), and involved lesional (PL) psoriasis skin. The expression values were obtained from NCBI GEO (Accession number: GSE13355). **(B)** Box plot showing the distribution of expression values of *TGM1*, *TGM2*, *TGM3*, and *TGM5* in PN and PL samples from *(A)*. **(C)** Analysis of the expression of *Tgm2* and *Tgm3* in murine skin treated with imiquimod. Expression values were obtained from NCBI GEO (Accession number: GSE27628). **(D)** Analysis of *TGM3* expression in normal and psoriatic skin. The expression values were obtained from NCBI GEO (Accession numbers: GSE6710, GSE14905, GSE78023, and GSE63741).

First, we investigated TG1, TG2, TG3, and TG5 mRNA expression levels in skin biopsies from 58 psoriasis patients, analyzing publicly available gene array data from the Gene Expression Omnibus (GEO) repository (Accession number: GSE13355). We observed a strong and significant upregulation in TG1 and TG3 expression in skin samples with involved lesional (PL) psoriasis (2- and 4-fold, *P* = 7 × 10^−43^ and *P* = 1 × 10^−26^, respectively) when compared to those of the involved non-lesional psoriasis (PN) and normal (N) skin samples. In the same array, no significant changes in TG2 and TG5 genes were observed in PL skin samples compared with those of PN and N skin samples. (Figure 1A,B). Since TG1KO mice are neonatal lethal [8], we then focused on TG3 to assess its role in psoriasis development. Additional analysis of human gene array data confirmed a significant increase in TG3 mRNA levels in human psoriatic skin samples (Figure 1D). We then expanded our TG3 bioinformatic analysis to an imiquimod-induced psoriasis model in C57BL/6 mice, using TG2 mRNA levels as a control, since TG2 expression is consistent within psoriasis-affected skin samples. As expected, a significant increase in TG3 mRNA levels was detected in murine psoriatic samples, whereas TG2 mRNA levels remained constant (Figure 1C).

### 2.2. Macroscopic Severity Markers of Psoriasis Display Similar Values in Imiquimod-Treated TG3KO, TG2KO, and Wild-Type Mice

**Figure 2:**
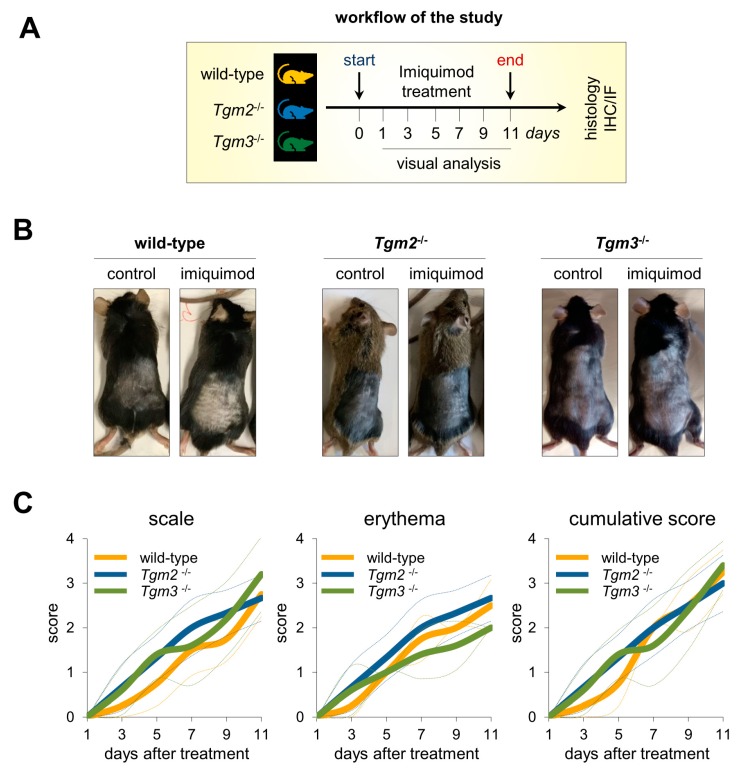
Imiquimod-treated TG3KO, TG2KO, and WT mice show similar macroscopic psoriasis severity grades. **(A)** Workflow of the study. **(B)** Photographs showing control and imiquimod-treated WT, TG2KO, and TG3KO mice. **(C)** Analysis of scale, erythema, and cumulative score during imiquimod treatment.

Topical application of imiquimod on the shaved back skin of C57BL/6 TG3KO and TG2KO mice for 10 days induced psoriasis-like lesions, such as redness, scales, desquamation, and crust formation (Figure 2A). As shown macroscopically in Figure 2B, the levels of desquamation and crusts were comparable in all imiquimod-treated TG3KO, TG2KO, and wild-type animals. Psoriatic skin from the three groups presented consistent grades in two out of three markers that are commonly utilized to grade psoriasis severity [39]. As shown in Figure 2C, the scores for erythema (redness), desquamation (scale), and their cumulative score for TG3KO mice during imiquimod application were not significantly different compared to the same psoriatic marker values for TG2KO or WT mice.

### 2.3. Imiquimod-Treated TG3KO Mouse Skin Shows Increased Epidermal Thickness and a Slightly Higher Grade of Basal Keratinocyte Proliferative Activity than Those of TG2-KO and WT Mice

**Figure 3:**
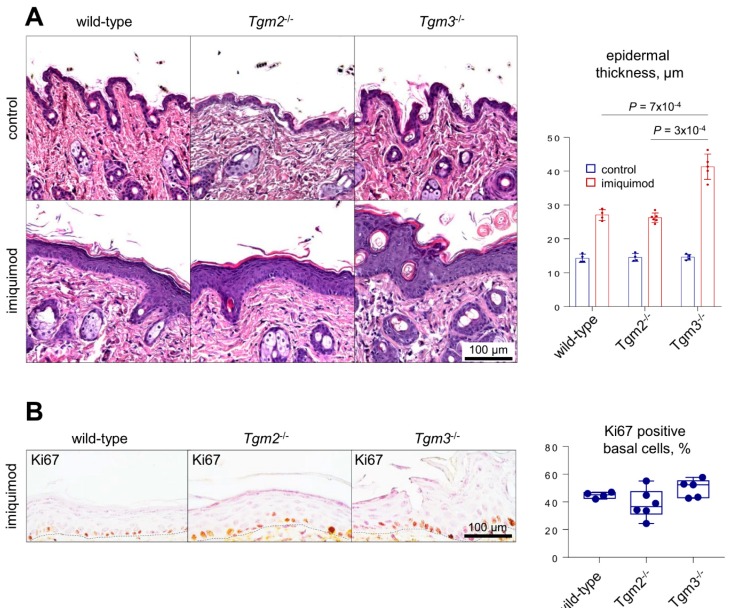
Loss of TG3 leads to epidermal thickening upon imiquimod treatment. Scale bar 100 μM. **(A)** H&E staining of the skin of either control or imiquimod-treated WT, TG2KO, and TG3KO mice. The graph shows the measurement of epidermal thickness. **(B)** Immunohistochemical staining for Ki67 in the skin of imiquimod-treated WT, TG2KO, and TG3KO mice. The graph shows the quantification of the percentage of Ki67-positive basal cells.

Histological evaluation of skin sections from wild-type, TG2KO, and TG3KO mice treated with imiquimod for ten days revealed characteristic alterations in psoriasis lesions, such as acanthosis (thickening of the epidermis), parakeratosis (presence of nuclei in the stratum corneum), desquamation, and dermal infiltrates. Interestingly, we observed a significant increase in the thickness of psoriatic TG3KO mouse epidermis (40 μM) compared to that of the TG2KO and WT psoriatic epidermis (<30 μM). Imiquimod application had increased qualitative and quantitative effects on TG3KO mice, including pustules, increases acanthosis, desquamation, and parakeratosis (Figure 3A). Imiquimod effects in the three animal groups were confirmed by IL-22 and IL-17a immunostaining, shown in Figure S1. 

Thus, imiquimod-treated mice presented apparent dysregulated hyperproliferation of keratinocytes compared to that of the controls in each group. Hyperproliferation of basal keratinocytes was also monitored by Ki67 immunohistochemical analysis. Ki67 is a cell proliferation marker, and in imiquimod-treated mice, Ki67 staining was slightly increased in the basal epidermal layer of TG3-KO mice compared to that of wild-type and TG2-KO mice, although the difference was not statistically significant (Figure 3B).

### 2.4. In TG3KO Mouse Epidermis, Imiquimod Treatment leads to Thickening of the Spinous Layer and to Reduced Organization of the Granular Compartment

**Figure 4:**
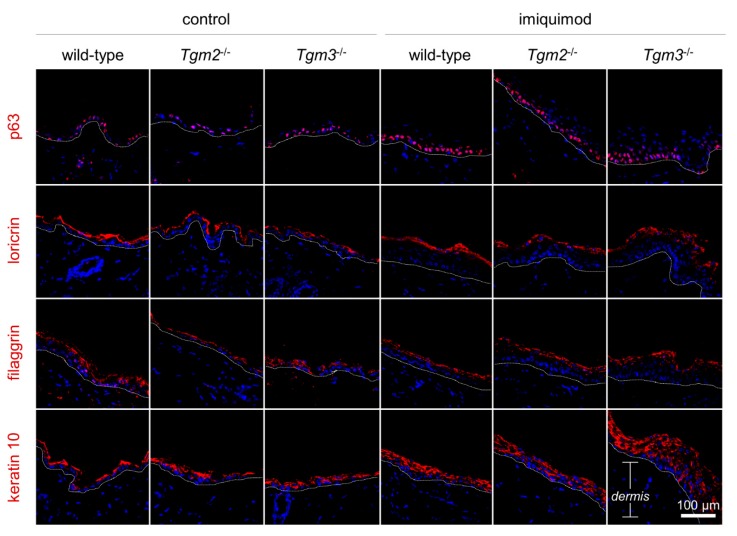
Imiquimod treatment leads to thickening of the spinous layer in the TG3KO mouse epidermis. Scale bar 100 μM. Immunofluorescence analysis of p63, loricrin, filaggrin, and keratin 10 expression in the skin of either control or imiquimod-treated WT, TG2KO, and TG3KO mice.

To evaluate the contribution of the different epidermal layers to the thickened epidermis observed in psoriatic TG3KO mouse skin, we performed immunofluorescence analysis of four epidermal differentiation markers, namely, p63, loricrin, filaggrin, and keratin 10, in the skin of controls and imiquimod-treated WT, TG2KO, and TG3KO mice. As shown in Figure 4, p63 protein, a marker of the proliferative compartment, was detected in the basal layers of all sections, while the two keratinocyte late differentiation markers filaggrin and loricrin were detected in the granular and upper granular compartments, respectively. In particular, we observed a more diffuse and disorganized distribution of loricrin in the psoriatic epidermis of TG3KO mice than in all other sections, probably due to a lack of TG3 crosslinking activity and to the consequent aberrant epidermal structure in these mice. Interestingly, imiquimod treatment led to a considerable thickening of the spinous layer in all of the treated mice, especially in TG3KO mice, as evidenced by the fluorescence labelling of keratin 10, a marker of the epidermal spinous layer. This observed hyperkeratosis might be the result of an increased permeability and sensitivity of TG3KO mouse skin to the psoriasis-inducing drug.

## 3. Discussion

The cornified envelope, a tight and membranous structure formed by highly crosslinked proteins and lipids, confers the unique characteristic of a strong barrier against physical, chemical, and microbial insults to the outermost layer of the epidermis. In the final stages of keratinocyte terminal differentiation, during CE generation, substrates such as loricrin, filaggrin, involucrin, trichohyalin, small proline-rich proteins, and keratins are crosslinked intracellularly by three transglutaminases (TG1, TG3 and TG5) [3]. Although it has been estimated that TG3 supports almost 75% of epidermal TG3 activity [40], its precise contribution to the synthesis of the CE has not yet been well defined in vivo. Indeed, except for impaired hair development, TG3KO mice do not show evident morphological abnormalities and have an apparently normal cutaneous structure and skin barrier [12]. However, the epidermal barrier of TG3KO mice appeared slightly dysfunctional upon challenge, including increased susceptibility to hapten FITC uptake or increased sensitivity to UV radiation [15,16]

TG2 is expressed in all epidermal layers, although its function in epidermal homeostasis is dispensable [21,22]. Dysregulation of pro-inflammatory TG2 activity elicits several inflammatory disorders [28,29,30,41]. TG2 is involved in activation of the inflammatory response in stressed tissues, where it stimulates both the release of inflammatory cytokines/chemokines and the recruitment of inflammatory cells into the stressed area [18,28,33]. In this study, we demonstrated that TG3 is necessary for proper cutaneous barrier formation and that its ablation in TG3KO mice increased epidermal susceptibility to the psoriasis-inducing drug imiquimod. We also confirmed previous data obtained in TG2KO mice, since TG2 gene deletion did not affect skin structure and resulted in indistinguishability from WT mice in the context of psoriasis. This latter result also suggests that TG2 is not involved in psoriasis-induced skin inflammation.

Psoriasis is an autoimmune and inflammatory skin disease that is triggered by a hyperactivated cellular immune system. Psoriatic cytokines, such as IL17, IFNγ, TNF-α, IL-22, and IL-23 are released from cells of the acquired and innate immune system in the dermis and stimulate inflammation, increased keratinocyte proliferation and aberrant keratinocyte differentiation that results in an inability to generate a proper skin barrier [42]. We suggest that the hyperexpression of TG1 and TG3 mRNA observed in our bioinformatic analysis of human psoriatic skin samples develops as a cause and/or a substantial compensation mechanism for the defective keratinocyte differentiation and hyperkeratosis observed in the inflamed psoriatic epidermis. Further database analysis confirmed increased TG1 and TG3 mRNA levels in imiquimod-induced mouse psoriatic skin samples, thus suggesting a probable beneficial dysregulation of these two genes in psoriasis. Conversely, TG5 and TG2 were unaffected by psoriatic inflammation, since their mRNA levels remained consistent in both psoriatic and normal skin biopsies. Our bioinformatic analysis was limited to mRNA levels only, and so no information was obtained regarding the detection of protein levels and/or their activities.

To elucidate the importance of TG3 and TG2 activities in the generation of a functional CE, we used the psoriatic imiquimod mouse model to test whether TG3KO mice and TG2KO mice display altered responses to the imiquimod drug compared with those of WT mice. When topically applied as a cream, imiquimod, a TLR7/8-stimulating compound, induces dermatitis that closely mimics psoriasis with typical hallmarks such as erythema, skin thickening, scaling, and epidermal alterations such as acanthosis and parakeratosis [38]. After 10 days of 5% imiquimod topical application on the shaved back skin, TG3KO, TG2KO, and WT mice, as expected, developed typical psoriatic plaques with markers that worsened with treatment time.

Grossly, psoriatic skin of TG3KO mice showed no macroscopic visual differences compared to those of TG2KO and WT mice. The severity of the scaling and erythema had comparable values and trends in the three groups throughout all treatment times, as displayed in their nearly overlapping graphs. This apparent lack of major changes in the skin when TG3 is ablated is in agreement with previous studies on TG3KO mice [12] and suggests that in the TG3KO epidermis, TG3 crosslinking activity is probably largely compensated for by the TG1 and TG5 enzymes. A more in-depth analysis at the histological level revealed a significantly thicker (+33%) epidermis in psoriatic TG3KO mice compared to that of WT and TG2KO mice. We hypothesize that the absence of TG3 protein generates an impaired and more fragile epidermal barrier structure that facilitates percutaneous absorption and penetration of imiquimod through the skin. In this regard, our results are in agreement with Bognar [15] and with our recent study on the more profound sensitivity of TG3KO skin to UV radiation [16]. It is likely that higher levels of absorbed imiquimod in TG3KO mice intensify hyperproliferation and abnormal keratinocyte differentiation as a cutaneous response to the psoriasis-inducing drug. Indeed, the thicker spinous layer and the more disorganized granular layer observed in immunofluorescence analysis of psoriatic TG3KO mouse skin are probably the result of a more pronounced incomplete keratinocyte differentiation that therefore halted at the level of the spinous layer and failed to become mature corneocytes. Loricrin is a TG3 substrate, and the observation of a diffuse pattern in TG3KO mice indicated a defective epidermal crosslinking in these mice. In our model, the expected increase in the hyperproliferative activity of keratinocytes in the basal layer of TG3KO mice was only modest, as detected by the number of Ki67-positive cells and visualization of immunofluorescence of p63 in the basal layer. This effect is probably due to the milder hyperproliferative power induced by the drug compared to the strong autoimmune effect of the spontaneous disease. Conversely, in our histological and immunofluorescence analyses, TG2KO skin sections were substantially indistinguishable from the corresponding WT skin sections in both imiquimod-treated and control samples. These data suggest that the inflammatory process associated with psoriatic disease is independent of TG2 expression or activation. Furthermore, the TG2KO results confirmed our recent studies in TG2KO mice whose keratinocyte morphological analysis and amino acid biochemical composition were very similar to those of WT mice and indicate that the activity of the TG2 enzyme is not necessary for CE assembly and the skin barrier [22].

## 4. Materials and Methods

### 4.1. Animal Studies

C57BL/6-TG2KO and C57BL/6-TG3KO mice were generated in our laboratory [21]. All mice used in this study were bred in the animal facility of the University of Roma “Tor Vergata” under specific pathogen-free conditions. All experiments were approved by the Institutional Animal Care and Use Committee (IACUC) and were carried out according to the Italian and European rules (D.L.116/92; C.E. 609/86; European Directive 2010/63/EU). For mice experiments licence n° 817/2016PR (Italian Ministry of Health). Male mice at 8 weeks of age were shaved using an electric shaver on the back skin and received topical applications of imiquimod (3.125 mg) from a commercially available cream (5%) daily for 10 days (Aldara; 3M Pharmaceuticals, St. Paul, MN, USA).

### 4.2. Scoring the Severity of Dermatitis

The degree of (1) erythema, (2) scaling, and (3) thickening was calculated as 0 (none), 1 (minor), 2 (moderate), 3 (severe), or 4 (very severe). The cumulative dermatitis grade (erythema plus scaling plus thickening) indicated the severity of dermatitis (scale 0–4).

### 4.3. Histology and Immunostaining

For haematoxylin and eosin staining, 3 µm FFPE (Formalin-Fixed Paraffin-Embedded) sections were dewaxed, rehydrated, stained in Mayer’s haematoxylin for 4 min, washed with tap water for 10 min, and then stained with eosin for 40 sec, followed by a rapid wash in water. The slides were dehydrated, mounted, and scanned using a 40× objective in a Ventana iCoreo scanner (Ventana, Oro Valley, AZ, USA). For Ki67 immunohistochemical staining, 3 µm FFPE sections were dewaxed, rehydrated, and blocked for 20 min in 0.3% hydrogen peroxide solution in methanol. Then, the slides were boiled in a microwave for 10 min in 0.01 M sodium citrate buffer (pH 6.0), blocked with 5% goat serum in PBS for 30 min, and incubated for 40 min with anti-Ki67 antibody (D2H10, 1:300, Cell Signaling, Leiden, Netherlands). The signal was detected using an UltraTek HRP anti-polyvalent DAB staining system (ScyTek, Logan, UT, USA), and then the slides were counterstained with haematoxylin for 10 sec, followed by a wash in tap water, dehydration, and mounting. IL-22 and IL-17A staining was performed using anti IL-22 antibody (NB100-733, 1:250, Novus Biologicals LLC, Centennial, USA) and IL-17A antibody (217359, 1:50, Abcam, Cambdridge, UK), respectively. For immunofluorescence staining, 10 µm cryosections were fixed in 4% paraformaldehyde for 10 min, permeabilized in a 0.1% PBS/Tween-20 solution for 10 min, blocked with 5% goat serum in PBS for 30 min, and incubated with primary antibodies overnight. The following antibodies were used: anti-p63 (D2K8x, 1:300, Cell Signaling), anti-Loricrin (Poly19051, 1:300, BioLegend, San Diego, CA, USA), anti-filaggrin (PRB-417P, 1:300, Covance, Princeton, NJ, USA), and anti-keratin 10 (PRB-159P, 1:1000, Covance). Then, the samples were washed and incubated with anti-rabbit AlexaFluor-568 antibody (1:1000, Invitrogen, Carlsbad, CA, USA) together with 1 μg/mL DAPI (Sigma, St. Louis, MO, USA) for nuclear DNA staining. Images were acquired by a Nikon A1 confocal laser microscope using NIS elements software (Nikon, Tokyo, Japan).

### 4.4. Bioinformatic Analysis

Normalized values of mRNA expression in the psoriasis-affected skin samples were obtained from the NCBI GEO portal (Accession numbers: GSE13355, GSE27628, GSE6710, GSE14905, GSE78023, and GSE63741).

### 4.5. Statistical Analysis

All statistical analyses were performed using GraphPad Prism 7.0 Software (San Diego, CA, USA). Two-tailed, unpaired Student’s t-tests were used for analysis of significant differences between two groups. Values of *p* < 0.05 were considered significant.

## 5. Conclusions

In the present study, we determined whether TG2 and TG3 are normally involved in the maintenance of skin barrier integrity and whether inflammatory processes modify the skin response to imiquimod-induced psoriasis. Our findings suggest that TG3 is crucial in the maintenance of a physiological cornified envelope in the stratum corneum and that its depletion sensitized mouse skin to the action of imiquimod, as demonstrated by H&E, IF, and Ki67 staining. In contrast, TG2KO mouse skin responded similarly to that of WT to imiquimod application, confirming the minor role of TG2 in skin barrier formation. Moreover, our data indicates, for the first time, that the pro-inflammatory properties of TG2 do not contribute to the onset of psoriatic disease, which is thus a TG2-independent process.

## Supplementary Materials

Supplementary materials can be found at https://www.mdpi.com/1422-0067/21/5/1566/s1.
