# Supplementary material for: Transglutaminase 3 Reduces the Severity of Psoriasis in Imiquimod-Treated Mouse Skin
Source: Int J Mol Sci. 2020 Feb 25;21(5):1566. doi: 10.3390/ijms21051566 (PMC7084269; doi:10.3390/ijms21051566)
Supplement: Supplementary file 1 [file ijms-21-01566-s001.pdf]

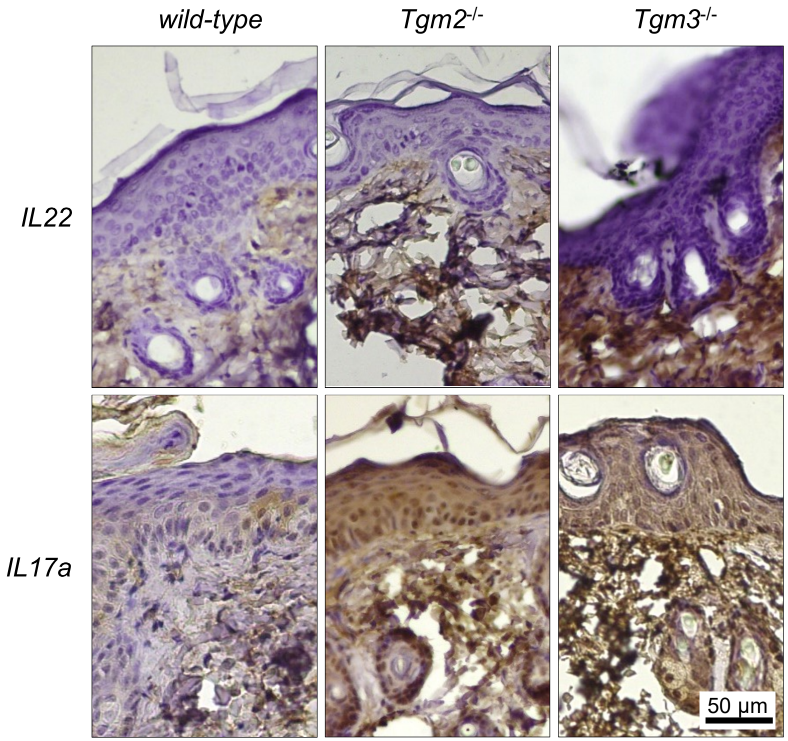

Figure S1. Loss of *Tgm3* leads to an increased IL-22 and IL-17A release upon imiquimod treatment. IL22 and IL17-A immunostaining in imiquimod treated wild-type, Tgm2$^{-/-}$ and Tgm3$^{-/-}$ mice skin samples confirmed inflammation induction *in vivo* and the increased effect of imiquimod on Tgm3$^{-/-}$ skin. One representative staining image/group is shown.

Figure S1 - Piro *et al*
